# Strain diversity in the microbiome: Lessons from *Bacteroides fragilis*

**DOI:** 10.1371/journal.ppat.1009056

**Published:** 2020-12-10

**Authors:** Hannah C. Carrow, Lakshmi E. Batachari, Hiutung Chu

**Affiliations:** 1 Department of Pathology, University of California San Diego, La Jolla, California, United States of America; 2 Chiba University-UC San Diego Center for Mucosal Immunology, Allergy, and Vaccine, La Jolla, California, United States of America; 3 Humans and the Microbiome Program, CIFAR, Toronto, Ontario, Canada; Tufts Univ School of Medicine, UNITED STATES

## Introduction

**Fig 1:**
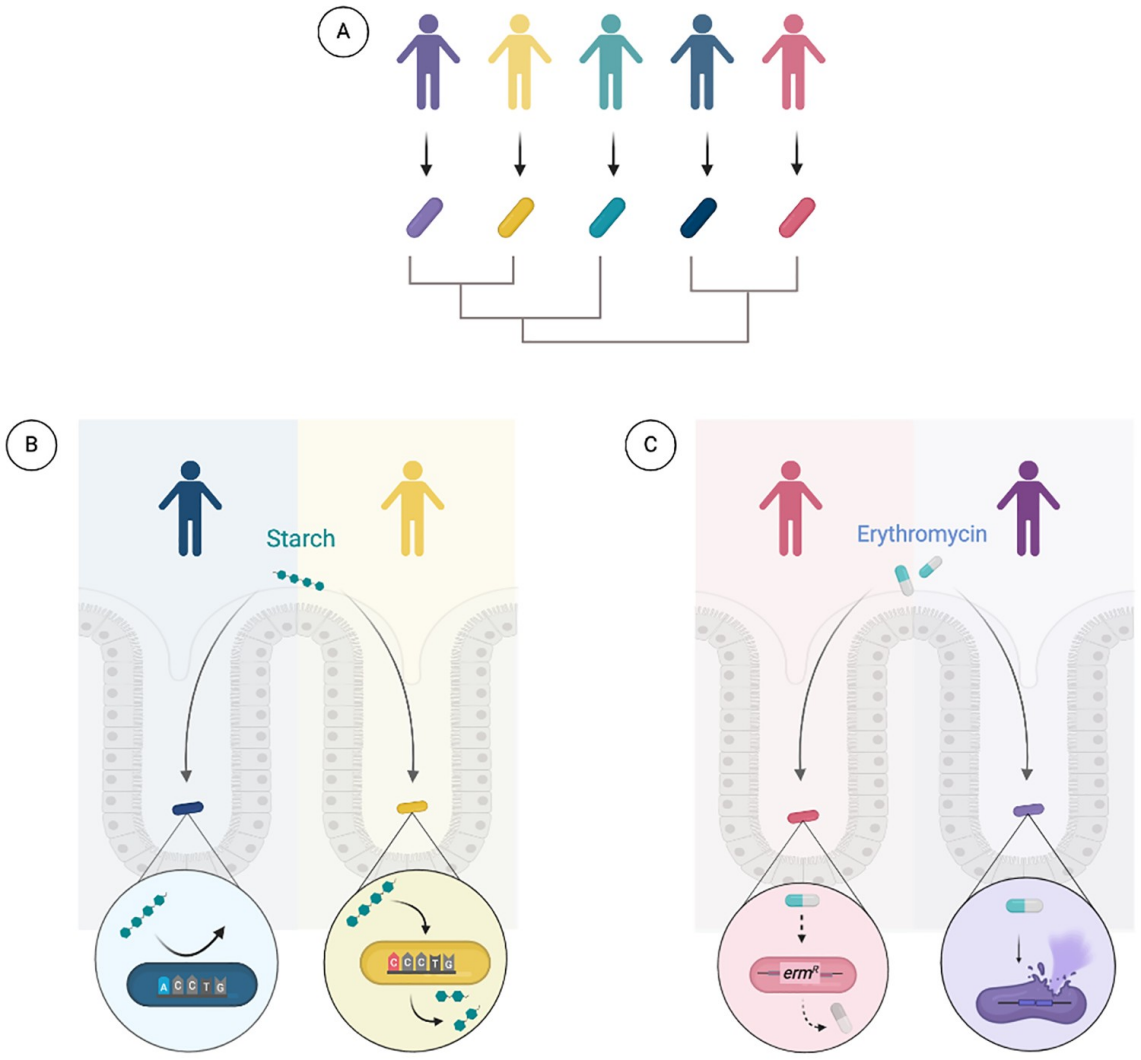
Host interactions with *Bacteroides fragilis* are strain-dependent. **(A)** Strains of *B*. *fragilis* vary across individuals. **(B)** *B*. *fragilis* relies on Sus-like systems to break down starch. Genetic variation in *sus* homologs may result from differences in host- and diet-derived glycans between individuals. **(C)** Increased exposure to antibiotics, such as erythromycin, can select for strains that have acquired resistance genes through horizontal gene transfer. Created with BioRender.com.

The evolutionary history between humans and their microbiota shapes a symbiotic relationship that is integral for host health. Culture-based approaches, animal studies, and advanced sequencing methodologies have unveiled the critical and unique influence of specific symbiotic microbes on host physiology. Even within a select species, strains exhibit significant variability in deriving host outcomes. The significance of strain diversity in the microbiome was well recognized more than 2 decades ago, as Abigail Salyers curated a collection of more than 200 isolates from the genus *Bacteroides* [1]. More recently, metagenomic sequencing and developments in computational approaches have enabled high-resolution analyses of bacterial strain diversity within a population [2–9]. Aided by these technologies, we now have the ability to elaborate how strain variability within a particular species yields functional diversity. In particular, the sequencing of new isolates of *Bacteroides fragilis* has resulted in an ever-expanding pangenome, revealing substantial genetic diversity within the species (Fig 1A) [9]. This genetic diversity is generated through genomic rearrangements, including inversions, duplications, and insertions via horizontal gene transfer (HGT) [1,10–12]. These processes constitute fundamental evolutionary mechanisms that promote genetic divergence and the emergence of novel bacterial functions. We are just beginning to understand how these strain-specific functions give rise to variable, and potentially individual-specific, host outcomes. Here, we use *B*. *fragilis* to discuss how the study of strain diversity in microbiome research can illuminate functional contributions of the microbiota in health and disease and uncover mechanisms of adaptation in the host gut.

## Studying *B*. *fragilis* strains to identify mechanisms of adaptation in the gut

Colonization with *B*. *fragilis* can begin in infancy and persist through adulthood. Studies on bacterial transmission suggest that *B*. *fragilis* is transferred vertically from mother to infant during vaginal delivery, or within the first year of life for infants born via cesarean section [5,13,14]. However, research on vertical transmission primarily utilizes species-level taxonomic assignment, whereas strain-level resolution is necessary to definitively determine modes of transmission [15]. Strain-level resolution can also highlight genes that contribute to adaptation within the gut. First described in *Bacteroides thetaiotaomicron*, SusC and SusD are involved in binding and transporting polysaccharides across the outer membrane [16,17]. Genetic analysis reveals recurrent mutations in *susC* and *susD* orthologs among *B*. *fragilis* strains isolated at different time points from the same individual [18]. This recurring mutation pattern was also detected in longitudinally sampled *B*. *fragilis* strains from other individuals. Variations in these genes may reflect adaptation to diet- or host-derived glycans (Fig 1B) [16]. Notably, mutations in the Sus homologs appear to be unique to each individual, suggesting microbial adaptation to host-specific factors [18]. As SusC and SusD are expressed on the cell surface, mutations in the genes encoding these proteins may allow for antigenic variation [16,17]. Strain variation in antigenicity may reflect a strategy that enables adaptation to and evasion of host immune processes, including immunoglobulin (Ig) recognition. *B*. *fragilis* strains can exhibit differences in the degree of IgA binding [19], which has been shown to influence mucosal colonization [20]. Other common targets of mutation included genes involved in the synthesis of the cell envelope. One of these genes, *ungD2*, is necessary for the synthesis of 7 of the 8 capsular polysaccharides located on the cell surface of *B*. *fragilis* [21]. This includes capsular polysaccharide A (PSA), which is well known for its immunomodulatory capabilities [22]. Mutations in genes associated with cell envelope biosynthesis, such as those described here, illustrate plausible adaptive mechanisms that allow bacteria to modulate and evade the host immune system. Analysis of strains that display divergence in glycan utilization and immune induction may unveil genetic factors that are critical for robust colonization within the host.

Strain-level analyses can also reveal host selective pressures and genes that are vital for adaptation at a population level. For example, strain-level metagenomic analysis of *B*. *fragilis* genomes identified a mutant allele of a gene that encodes a predicted periplasmic protein. The missense mutation was prevalent in a Western cohort but absent in a Chinese cohort [18]. This discrepancy implies that *B*. *fragilis* strains face distinct host selective pressures dependent on diet, lifestyle, and host genetics. Future studies can determine the potential functional impact of the observed mutation [18], which may explain the relationship between mechanisms of adaptation and selective pressures. Selective forces can also arise from differences in antibiotic usage between populations. Longitudinal and geographical analyses of *B*. *fragilis* isolates reveal disparities in antibiotic resistance among strains. Erythromycin and tetracycline resistance genes were more frequent in strains isolated after 1980 compared to those isolated prior to 1970, reflecting an increase in antibiotic consumption within the population (Fig 1C) [1]. Geographically, imipenem resistance was more prevalent among *B*. *fragilis* isolates from Japan [23], where imipenem was prescribed at higher rates, compared to strains from Europe isolated at approximately the same time period [24]. Experimental work found that the multidrug resistant strain *B*. *fragilis* HMW615 was able to transfer antibiotic resistance genes to *B*. *fragilis* 638R, revealing a possible mechanism by which *B*. *fragilis* adapts to the dynamic gut environment [12]. By studying strains from diverse populations, we can begin to uncover mechanisms of adaptation and track evolutionary trends that can inform research and clinical decisions.

## Studying *B*. *fragilis* strains to elucidate their functional contributions to health and disease

*B*. *fragilis* can play a dichotomous role in its interactions with the host. The non-toxigenic *B*. *fragilis* (NTBF) type strain NCTC 9343 directs a tolerogenic immune response, suppressing intestinal inflammation in mice [22,25]. However, enterotoxigenic *B*. *fragilis* (ETBF) strains drive inflammation of the colon and are associated with colorectal cancer [26–29]. A distinguishing feature of ETBF is *B*. *fragilis* toxin (BFT), a metalloprotease that causes barrier disruption and intestinal inflammation [28,30]. BFT is encoded within a genome segment, the *B*. *fragilis* pathogenicity island (BfPAI), that is flanked by genes encoding mobilization proteins (CTn86, a conjugative transposon) [31]. These mobile elements suggest that the BfPAI is transmissible, which supports evidence that the genome segment was independently acquired multiple times, akin to strategies of acquiring antibiotic resistance genes [32]. Independent acquisition of BfPAI by NTBF strains may explain the finding that ETBF and NTBF strains do not cluster as 2 monophyletic groups. Further, sequence analysis revealed NTBF 638R is more closely related to an ETBF strain than another NTBF strain, NCTC 9343 [29]. The functional capabilities of *B*. *fragilis* strains likely reflect a continuum instead of a dichotomy.

Although ETBF is associated with disease, up to 30% of individuals who harbor ETBF do so asymptomatically [28]. This discrepancy suggests that ETBF strains may vary in pathogenicity, although host susceptibility may also play a role [33]. Differences in pathogenic potential may be attributed to variation in BFT production across ETBF strains [34]. Screening a diverse population of ETBF isolates at the genetic and functional level may point to genes and mechanisms that are responsible for this variable BFT production. Moreover, ETBF strains can harbor different *bft* isotypes (*bft-1*, *bft-2*, *bft-3*) [28]. The presence and copy number of a given *bft* isotype may contribute to the seemingly variable pathogenicity among ETBF strains [34]. For example, BFT-2 is associated with colorectal cancer and exhibits greater carcinogenic potential than BFT-1 [35,36]. In HT-29 cells, strains with *bft-1* and *bft-2* exhibit slightly higher cytotoxicity compared to strains containing *bft-3* [34]. Because differences in cytotoxicity are subtle, comparing the functional activities of all 3 BFT isoforms in a model system that is skewed toward inflammation may inform whether a given isotype exacerbates disease. Currently, limited data exists to establish a causal relationship between *bft* variants and chronic inflammatory diseases.

Adding even more complexity, BFT may not be the only virulence factor in ETBF. Within the BfPAI, *mpII* encodes for another metalloprotease with potential pathogenic properties [32]. In a germ-free mouse model, both a wild-type NTBF strain that overexpressed BFT and an ETBF strain were able to induce colitis. Yet, the BFT-expressing NTBF strain did not induce inflammation to the same extent as the ETBF strain [27]. Variation in biofilm formation, which does not require *bft*, could contribute to disparities in pathogenicity [29]. Compared to NTBF strains, ETBF strains demonstrate increased biofilm activity, which can confer resistance to antimicrobials and strengthen adherence to host epithelial cells [29]. The contributions of ETBF to disease are multifaceted. Strain-level resolution can identify why ETBF induces inflammation in some individuals but not in others, or the extent to which BFT isoforms and other pathogenic determinants contribute to the progression of diseases, such as colorectal cancer.

Despite its associations with disease, *B*. *fragilis* also promotes immune tolerance in the gut. The type strain *B*. *fragilis* NCTC 9343 expresses PSA, which drives regulatory T cells to produce interleukin-10, an anti-inflammatory cytokine responsible for maintaining immune tolerance [22,25]. It remains unclear if all or most *B*. *fragilis* strains promote this PSA-dependent immune response, but varied host responses may arise from variation in the expression and structure of PSA among strains. In a study of 50 diverse *B*. *fragilis* strains, the regions flanking the PSA locus were found to be conserved [37]. However, the central portion of the PSA locus was not conserved among all 50 strains, revealing heterogeneity among *B*. *fragilis* PSA. The commonly used lab strain *B*. *fragilis* 638R exhibited a distinct PCR product within the PSA locus [37], raising the possibility that 638R is not phenotypically representative of *B*. *fragilis* in the human population. Of note, recent genetic comparisons reveal that the PSA locus of NCTC 9343 is not conserved in *B*. *fragilis* HMW615 or *B*. *fragilis* 638R [12].

The extensive variation in the *B*. *fragilis* PSA locus suggests variability in PSA structure [38]. Consistent with the distinct PSA locus described above, the PSA structure derived from 638R is more complex, with 5 monosaccharides instead of the 4 in the PSA of NCTC 9343 [38,39]. Outside of the intestinal environment, where *B*. *fragilis* is associated with inflammation, the PSA of *B*. *fragilis* NCTC 9343 was a more potent inducer of peritoneal abscess formation in a rat model compared to the PSA of *B*. *fragilis* 638R [40]. Within the gastrointestinal tract, *B*. *fragilis* is capable of suppressing inflammation. Therefore, PSA variation among strains may influence the induction of a tolerogenic response required for maintaining homeostasis [11,12,37]. The PSA-driven response among *B*. *fragilis* strains warrants further study, given that the relationship between the host immune system and NTBF is strain-dependent.

Common *B*. *fragilis* lab strains, including NCTC 9343 and 638R [11], were originally isolated from infections, yet we rely on them to study *B*. *fragilis* in the context of immune tolerance in the gut [22,25]. Clinical isolates may induce a distinct immune response compared to fecal isolates [41]. For example, in mice with LPS-induced inflammation, *B*. *fragilis* NCTC9343 did not significantly affect the production of pro-inflammatory cytokine tumor necrosis factor alpha (TNF-α), whereas a *B*. *fragilis* strain isolated from a healthy donor, *B*. *fragilis* HCK-B3, down-regulated TNF-α [41]. Further, *B*. *fragilis* strains can exhibit varied sensitivity to immune mediators in the gut. Fecal isolates are more susceptible to the antimicrobial peptide, human β-defensin-3, compared to blood and extraintestinal isolates [42]. By evading host antimicrobials, which are critical factors of immune homeostasis, certain *B*. *fragilis* strains may be more likely to translocate out of intestinal tissue and cause disease. The intestinal environment, in either healthy or pathologic conditions, shapes the adaptive strategies of bacterial strains. Over the course of our life span, the changing gut environment (e.g., diet, medication, and immune status) presents opportunities for new strains to evolve, driving genomic and functional diversity.

## Conclusions and future perspectives

Emerging technologies have illuminated the rich diversity of bacterial strains in the human microbiome. *B*. *fragilis* serves as an example to illustrate the range of phenotypic heterogeneity within a commensal species. By studying bacterial strains across different populations, we can gain insight into genes and functions that are under selection in the human microbiota. For instance, how might chronic inflammatory conditions drive strain selection and divergence? We predict that prolonged inflammation in a sick individual may give rise to *B*. *fragilis* strains that are distinct from those found in a healthy individual, but further work is needed to validate this notion. On the other hand, how might strain diversity influence disease outcomes? Deeper examination of bacterial strains associated with disease may uncover pathological phenotypes driven by select strains. For example, genomic analysis revealed that specific *B*. *fragilis* strains were associated with type 2 diabetes [4]; however, functional studies are required to determine if these strains are the cause or effect of disease. The need for strain-level analysis is not limited to *B*. *fragilis*. Strains of gut commensal *Eggerthella lenta* show differences in the ability to inactivate the cardiac drug digoxin, which can affect clinical outcomes [43]. Moreover, *Prevotella copri* strains from Western populations and non-Western populations show significant genetic differences [33]. The unprecedented characterization of commensal strain diversity presents us with a compelling opportunity to ask fundamental questions about the relationship between us and our microbiota. This perspective serves to highlight the functional implications of bacterial strain variability and to illustrate how strain-level research can draw causal connections between the microbiome and disease.
